# A Fast, Open EEG Classification Framework Based on Feature Compression and Channel Ranking

**DOI:** 10.3389/fnins.2018.00217

**Published:** 2018-04-16

**Authors:** Jiuqi Han, Yuwei Zhao, Hongji Sun, Jiayun Chen, Ang Ke, Gesen Xu, Hualiang Zhang, Jin Zhou, Changyong Wang

**Affiliations:** ^1^Department of Neural Engineering and Biological Interdisciplinary Studies, Institute of Military Cognition and Brain Sciences, Academy of Military Medical Sciences, Beijing, China; ^2^College of Life Science and Technology, Huazhong Agricultural University, Wuhan, China; ^3^Neural Interface & Rehabilitation Technology Research Center, Huazhong University of Science and Technology, Wuhan, China

**Keywords:** EEG classification, channel selection, feature clustering, EEG low-dimensional representation, motor imagery

## Abstract

Superior feature extraction, channel selection and classification methods are essential for designing electroencephalography (EEG) classification frameworks. However, the performance of most frameworks is limited by their improper channel selection methods and too specifical design, leading to high computational complexity, non-convergent procedure and narrow expansibility. In this paper, to remedy these drawbacks, we propose a fast, open EEG classification framework centralized by EEG feature compression, low-dimensional representation, and convergent iterative channel ranking. First, to reduce the complexity, we use data clustering to compress the EEG features channel-wise, packing the high-dimensional EEG signal, and endowing them with numerical signatures. Second, to provide easy access to alternative superior methods, we structurally represent each EEG trial in a feature vector with its corresponding numerical signature. Thus, the recorded signals of many trials shrink to a low-dimensional structural matrix compatible with most pattern recognition methods. Third, a series of effective iterative feature selection approaches with theoretical convergence is introduced to rank the EEG channels and remove redundant ones, further accelerating the EEG classification process and ensuring its stability. Finally, a classical linear discriminant analysis (LDA) model is employed to classify a single EEG trial with selected channels. Experimental results on two real world brain-computer interface (BCI) competition datasets demonstrate the promising performance of the proposed framework over state-of-the-art methods.

## 1. Introduction

For a long time, the brain-computer interface (BCI) has been a prevalent communication system for directly bridging between a brain and a computer. A typical BCI system collects brain activities associated with mental tasks, translates these neural signals into appropriate commands, and eventually sends them to a computer (Handiru and Prasad, 2016). As a non-invasive brain activity measurement method, electroencephalography (EEG) has attracted increasing interest, owing to its low risk, low cost, feasibility, and significant potential for practical applications (Yang et al., 2016). EEG data is commonly composed of multichannel signals recorded from several electrodes placed on the scalp to obtain the activity of various cortexes. For example, motor-imagery (MI) EEG signals are primarily gained from the motor-relevant cortex and could provide users with direct control of various devices (Lafleur et al., 2013; Meng et al., 2016), e.g., a wheelchair, quadcopter, or robotic arm, without using any peripheral nerves or muscle movements.

The core component of an EEG-based BCI (EEG-BCI) system is the decoding or classification of EEG signals. The basic structure of an EEG classification framework typically consists of four parts: signal pre-processing, feature extraction, channel selection and classification, of which the feature extraction and channel selection are attracting more attention. Feature extraction is fundamental to EEG classification and many methods have been presented to acquire the implicit essential information from raw EEG signals, e.g., spectral power (SP) and time-domain parameters (TDP) (Müller et al., 2004). Channel selection attempts to remove irrelevant EEG features in redundant channels to reduce the setup time and equipment cost, and improve the effectiveness and efficiency of EEG-BCI systems (Alotaiby et al., 2015). Well-known channel selection methods are often based on evolutionary algorithms and mutual information.

**Figure 1:**
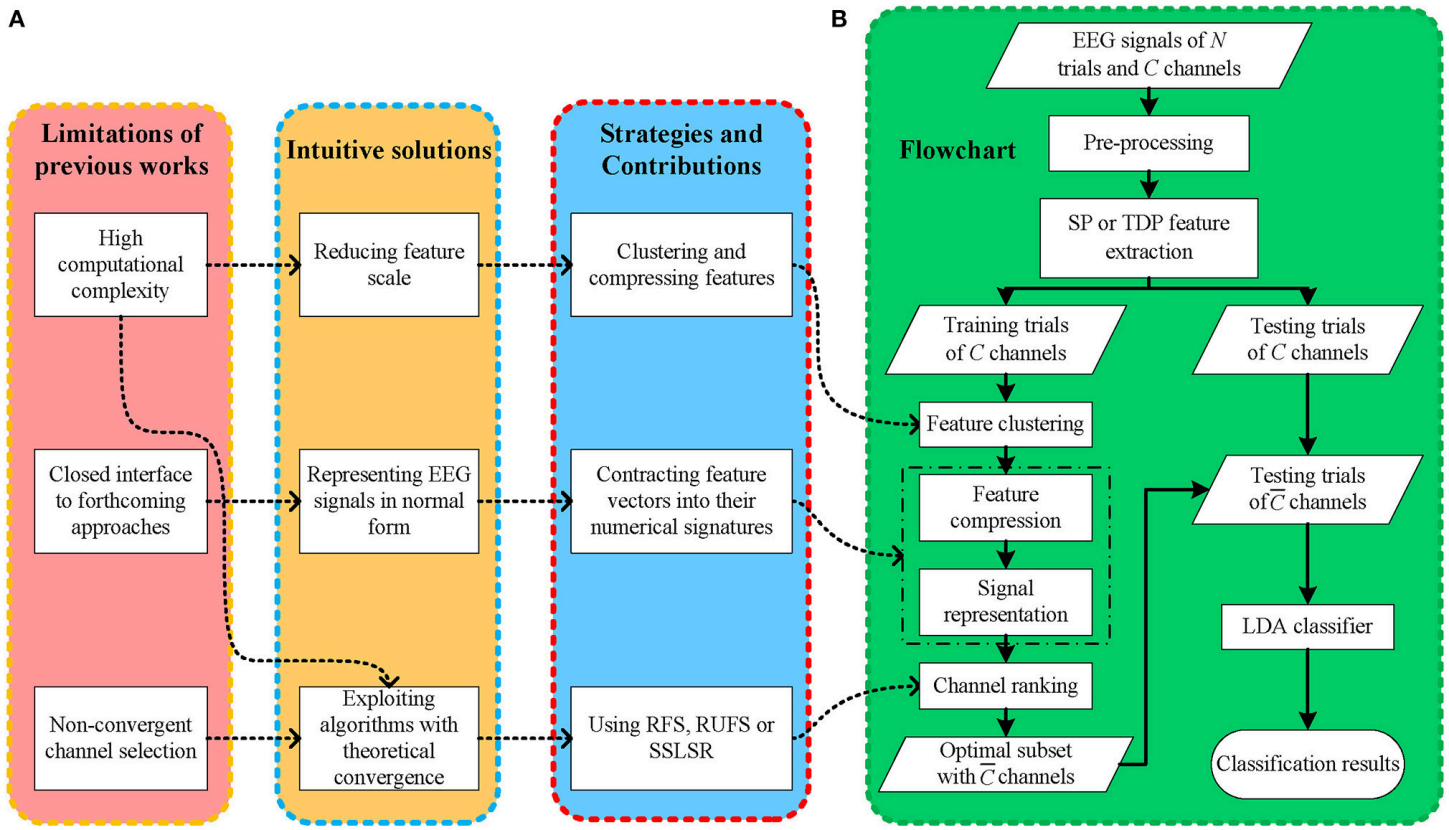
**(A)** The motivations, solutions and contributions, **(B)** the flowchart of the proposed framework.

Even though multiple methods have been proposed to tackle EEG classification tasks using different feature extraction and channel selection methods, bottlenecks still appear when existing EEG-BCI frameworks are applied to practical applications, as shown in Figure 1A. (1) The computational complexity of channel selection methods has often been neglected by previous works (Kee et al., 2015; Zhang et al., 2016), thereby slowing the EEG feature extraction and offline classifier training procedures, which negatively affects the deployment efficiency of EEG frameworks in different situations. Moreover, excessively long setup and experiment times may negatively affect the (human) subjects' mental concentration and condition, heavily decreasing the EEG signal-to-noise ratio (SNR). (2) Convergence is seldom considered in some iterative frameworks (Yu et al., 2015), leading to stochastic, even divergent solution searching. A non-monotonic convergence process may postpone the EEG classification and further degenerate the subjects' experience. Moreover, it is difficult to consistently obtain an optimal decision persistently because of the unstable solution searching process. (3) Existing frameworks are mostly designed too specifically (Bashar et al., 2016), narrowing their expansibility. The majority of datasets in pattern recognition approaches are expressed in two-dimensional matrices, i.e., samples and features, which differ from previous EEG data matrices. Thus, an overly well-designed EEG framework is usually confined to specific methods and offers few open accesses to the many more remaining methods, thus, seriously affecting its application and expansion. Additionally, considering that novel methods are continuously emerging, inadequate support for them may limit the sustained improvement of existing frameworks.

Intuitively, the solution to (2) is to use fast convergent channel selection algorithms, which is also beneficial for solving (1). Another solution to (1) is decreasing the data scale, including feature extraction and dimension reduction. Meanwhile, the solution to (3) is to present the EEG signals in two dimensional data matrices, which are commonly used in other signal processing areas, without removing the latent spatial information carried by multi-channel EEG signals. In the meantime, representing EEG data in two dimensional matrices is conducive to building bridges between EEG feature extraction and previous feature selection methods.

In light of this, as shown in Figure 1A, we propose a fast, open EEG classification framework characterized by feature compression, low-dimensional representation, and convergent iterative channel ranking. Firstly, to alleviate the computational burden, we use fast data clustering to give EEG feature vectors numerical signatures according to their channel-wise similarity. Thus, it is possible to reduce the feature dimension in EEG signals. Secondly, instead of simply flattening three dimensional (trial-channel-feature) EEG signals into two dimensional (trial-channel^*^feature) ones, we compress the EEG features and contract the feature vectors into their numerical signatures. Thus, we can represent the EEG signal using a two dimensional matrix and retain its spatial information in the meantime. In this manner, EEG signals are mapped into a normal data matrix with rows and columns indicating trials and channels, respectively, which is consistent with most pattern recognition studies. Thirdly, to obtain fast and stable decisions, we use a few iterative feature selection approaches with theoretical convergence to rank and select informative EEG channels, thereby shortening the procedure and improving the decision performance of the EEG-BCI systems.

Our main aims and contributions are highlighted in the following:

- We leverage the data clustering between feature extraction and channel selection in the traditional EEG-BCI to channel-wise compress the features to reduce the data scale.
- We provide an EEG classification framework applicable to most common EEG processing and pattern recognition methods to provide an entrance for increasing the EEG-BCI performance through technology integration.
- We introduce feature selection approaches with theoretical convergence to rank and select channels to further improve the performance of the EEG-BCI.

## 2. Related work

In past decades, great efforts have been made to address the EEG classification problem, mainly including feature extraction and channel selection.

One of the most widely used EEG feature extraction methods is common spatial patterns (CSP). CSP utilizes covariance analysis to amplify the class disparity in the spatial domain, to combine signals from different channels (Blankertz et al., 2007; Li et al., 2013). Improved CSPs, e.g., common spatio-spectral patterns (CSSP) (Lemm et al., 2005), iterative spatio-spectral pattern learning (ISSPL) (Wu et al., 2008), and filter bank common spatial patterns (FBCSP) (Kai et al., 2012), mainly optimize the combination of multi-channel signals by developing a spectral weight coefficient evaluation. Obviously, these spatial feature extraction methods are good at selecting the pivotal spatial information included in EEG signals. However, signal combination cannot be practically used to reduce the channel number and data scale. Apart from the spatial domain, extracting features in the temporal and frequency domains is also prevalent in many works. Multivariate empirical mode decomposition (MEMD) generates multiple dimensional envelopes by projecting signals in all directions of various spaces (Rehman and Mandic, 2010; Islam et al., 2012). The autoregressive (AR) model assumes that EEG signals can be approximated in the AR process, in which the features could be gained as parameters of the approximated AR models (Zabidi et al., 2013). TDP was introduced as a set of broadband features based on the variance of different EEG signals in various orders (Vidaurre et al., 2009). A wavelet transform (WT) simultaneously provides ample frequency and time information about EEG signals at the low and high frequencies, respectively (Jahankhani et al., 2006). We utilize SP based on AR and TDP as two feature extraction examples in this paper.

EEG channel selection has been extensively studied. For instance, multi-objective genetic algorithms (GA) (Kee et al., 2015) and Rayleigh coefficient maximization based GA (He et al., 2013) were introduced to simultaneously optimize the number of selected channels and improve the system accuracy by embedding classifiers into the GA process. Recursive feature elimination (Guyon and Elisseeff, 2003) and zero-norm optimization (Weston et al., 2003) based on the training of support vector machines (SVMs) were used to reduce the number of channels without decreasing the motor imagery EEG classification accuracy (Lal et al., 2004). Sequential floating forward selection (SFFS) (Pudil et al., 1994) and successive improved SFFS (ISFFS) (Zhaoyang et al., 2016) took an iterative channel selection strategy that selected the most significant feature from the remaining features and dynamically deleted the least meaningful feature from the selected feature subset. A Gaussian conjugate group-sparse prior was incorporated into the classical empirical Bayesian linear model to gain a group-sparse Bayesian linear discriminant analysis (gsBLDA) method for simultaneous channel selection and EEG classification (Yu et al., 2015). Mean ReliefF channel selection (MRCS) adopted an iterative strategy to adjust the ReliefF-based weights of channels according to their contribution to the SVM classification accuracy (Zhang et al., 2016). The Fisher criterion, based on Fisher's discriminant analysis was utilized to evaluate the discrimination of TDP features, extracted from all channels in different time segments via channel selection, using time information (CSTI) methods (Yang et al., 2016). GA and pattern classification using multi-layer perceptrons (MLP) and rule-extraction based on mathematical programming were combined to create a generic neural mathematical method (GNMM) to select EEG channels (Yang et al., 2012). However, a visible limitation is that the convergence of most of these methods is unstable, e.g., gsBLDA, leading to an uncertain channel selection procedure and selected subset.

Therefore, it is natural to select EEG channels by taking advantage of feature selection methods in other areas, e.g., robust feature selection (RFS) (Nie et al., 2010), joint embedding learning and sparse regression (JELSR) (Hou et al., 2011), nonnegative discriminative feature selection (NDFS) (Li et al., 2012), selecting feature subset with sparsity and low redundancy (FSLR) (Han et al., 2015b), robust unsupervised feature selection (RUFS) (Qian and Zhai, 2013), joint Laplacian feature weights learning (JLFWL) (Yan and Yang, 2014), a general augmented Lagrangian multiplier (FS_ALM) (Cai et al., 2013) and structural sparse least square regression based on the *l*_0_-norm (SSLSR) (Han et al., 2015a). RFS, JELSR, and NDFS focused on the *l*_2, 1_-norm minimization regularization to develop an accurate and compact representation of the original data. RUFS tried to solve the combined object of robust clustering and robust feature selection using a limited-memory BFGS based iterative solution. JLFWL selected important features based on *l*_2_-norm regularization, and determined the optimal size of the feature subset according to the number of positive feature weights. FSLR could retain the preserving power, while implementing high sparsity and low redundancy in a unified manner. FS_ALM and SSLSR attempted to handle the least square regression based on *l*_0_-norm regularization by introducing a Lagrange multiplier and a direct greedy algorithm, respectively. Further, prevalent deep learning was also utilized in this area. A point-wise gated convolutional deep network was developed to dynamically select key features using a gating mechanism (Zhong et al., 2016). Multi-modal deep Boltzmann machines were employed to select important genes (biomarkers) in gene expression data (Syafiandini et al., 2016). A deep sparse multi-task architecture was exploited to recursively discard uninformative features for Alzheimer's disease diagnosis (Suk et al., 2016). In this paper, we employ three performance-verified feature selection methods with theoretical convergence, i.e., RFS, RUFS, and SSLSR, to rank and select channels.

## 3. Materials and methods

In this section, we describe the datasets, introduce some notations used throughout this paper, and present the proposed frameworks.

### 3.1. Datasets and pre-processing

In our experiments, we selected two public real world datasets on motor imagery (MI) paradigms as examples. A brief description of these two datasets is offered below and their basic statistics are summarized in Table 1. EEG datasets of other paradigms, e.g., P300, are also suitable for this framework.

**Table 1:** Statistics of the two datasets.

**Datasets**	**DS1**	**DS2**
Number of channels	118	59
Sampled frequency (Hz)	100	100
Number of subjects	5	4
Subjects' name	aa,al,av,aw,ay	a,b,f,g
Number of trials per class	140	100

#### 3.1.1. DS1

Dataset IVa from BCI Competition III is a public EEG dataset provided by the Berlin BCI group Fraunhofer FIRST (Intelligent Data Analysis Group) and Campus Benjamin Franklin of the *Charité* University (Neurophysics Group). This public dataset is recorded from five healthy subjects (aa,al,av,aw,ay) during right hand and right foot motor imageries. The EEG recordings consist of 118 channels at positions of the extended international 10/20-system. We choose a version of the data that is down-sampled at 100 Hz for analysis. In the experiments, subjects performed three motor imageries for 3.5 s after visual cues for left hand, right hand, or right foot. After the duration of motor imagery, a resting interval with random length of 1.75–2.25 s was inserted for relaxation. The dataset provides only EEG trials for right hand and right foot imagery. For each subject, the dataset consists of signals of 140 trials per class.

In our experiment, the signals in the time interval of [0.5, 3.0] s are analyzed for each trial. A visual cue is presented to mark the start time (0 s). A bandpass finite impulse response (FIR) filter using the window method, with a band of 0.1–40 Hz and order 33, is applied to DS1.

#### 3.1.2. DS2

Dataset I from BCI Competition IV is another public EEG dataset provided by the Berlin BCI group Fraunhofer FIRST (Intelligent Data Analysis Group) and Campus Benjamin Franklin of the *Charité* University (Neurophysics Group). This public dataset is recorded from four healthy subjects (a,b,f,g) during two classes of motor imagery selected from three classes: left hand, right hand, and foot (side chosen by the subject; optionally also both feet). In the experiments, the data was continuous signals of 59 EEG channels and visual cues pointing left, right or down were presented for a period of 4.0 s during which the subject was instructed to perform the cued motor imagery task. These periods were interleaved with 2.0 s of blank screen and 2.0 s with a fixation cross displayed in the center of the screen. The dataset provides only EEG trials for left hand and foot imagery. For each subject, the dataset consists of signals of 100 trials per class.

In our experiment, the signals in the time interval of [0.0, 4.0] s are analyzed for each trial. A visual cue is presented to mark the start time (0 s). A bandpass FIR filter using the window method, with a band of 0.1 to 40 Hz and order 33, is also applied to DS2.

### 3.2. Notations

In this document, scalars, matrices, vectors, sets, and functions are denoted as small, boldface capital, boldface lowercase, blackboard capital, and script capital letters, respectively. **x**^*T*^, **X**^*T*^, **x**_*i*_, **X**_*i*_, **X**_*ij*_, **X**_(*i*,:)_, **X**_(:, *j*)_, and trace(**X**)indicate the transpose of vector **x**, the transpose of matrix **X**, the *i*-th element of **x**, the *i*-th sample of the variable **X**, the element of **X** occurring in the *i*-th row and *j*-th column, the *i*-th row of **X**, the *j*-th column of **X** and the trace of **X** respectively. Moreover, ||**x**||_1_ is the *l*_1_-norm of **x**, ||**X**||_1_ and ||**X**||_2_ are the *m*_1_-norm and *m*_2_-norm of matrix **X**. For any vector **x** ∈ ℝ^*n*×1^ and any matrix **X** ∈ ℝ^*n*×*m*^, the definitions of *l*_*p*_-norm, *m*_*r*_ -norm and *m*_*r, s*_ -norm are given in the Appendix.

Furthermore, assume that we have recorded EEG signals of *N* trials, and let $𝕏={\left\{{\text{X}}_{i}\right\}}_{i=1}^{N}$ be the set of EEG signal corresponding to all *N* trials. Specifically, we represent each trial of EEG signals, i.e., **X**_*i*_(*i* = 1, 2, …, *N*), as matrix **X**^*M*×*C*^, where *M* and *C* are the number of sampled time points and channels in a trial respectively. The class indicator vector can be denoted as **y** ∈ {0, 1, …, *L* − 1}^*N*^, where *L* is the number of classes.

### 3.3. Proposed methods

We first depict the flowchart of the framework, called feature compressing and channel ranking (FCCR). Afterward, we provide the details for its three core components. Lastly, its pseudo codes are given.

#### 3.3.1. Flowchart of the framework

Our framework starts with EEG signal pre-processing and single-channel SP or TDP feature extraction. Next, after dividing all of the trials into the training and testing sets, we gather all the features from the different channels for the training trials and assign them cluster signatures through *k*-means. Then, EEG signals are mapped from the three dimensional (trial-channel-feature) to the two dimensional (trial-channel) matrix by compressing all feature vectors into their cluster signatures. With this data representation (two-dimensional matrix) commonly used in most pattern recognition areas, we employ RFS, RUFS, or SSLSR to rank and select the channels. Lastly, a traditional LDA is used to classify the testing trials, with the features corresponding to the selected channels. The procedure of our proposed FCCR is summarized in Figure 1B. Notably, FCCRs are subject-specific since the procedures from feature clustering to channel ranking in Figure 1B are dependent of the subject.

#### 3.3.2. Feature extraction

We take SP based on AR model and TDP as two examples to extract EEG features. In fact, other methods, including WT and MEMD, can also be used to replace them.

Each EEG signal of a single channel is treated as a time-varying variable, denoted as **x**(*t*), in this section.

##### 3.3.2.1. SP features

A power spectrum is an energy density distribution over frequencies. The AR Filter actually computes a time series of such spectra, representing a flow of energy through each single point in the frequency domain. In this way, the SP based on AR model can be treated as the energy per frequency per time.

AR model is a representation of a type of random process (Zabidi et al., 2013), which specifies that the output variable depends linearly on its own previous values. Hence, the predicted value $\hat{\text{x}}\left(t\right)$ of an AR process is defined by,

(1) $$\begin{array}{l} \hat{\text{x}}\left(t\right)=-\overset{p}{\underset{k=1}{\sum }}a_{p}\left(k\right)x\left(t-k\right), \end{array}$$

where *a*_*p*_(*k*) is the coefficient of the *p*-th order AR process.

Then, the estimated power spectrum directly corresponds to the filter's transfer function whose coefficients are actually AR coefficients. To obtain spectral power for finite-sized frequency bins, that power spectrum is multiplied by total signal power, and integrated over the frequency ranges corresponding to individual bins. We select frequency bins from 0 to 30 Hz with step 3 Hz, resulting 11 values per channel for each trial.

##### 3.3.2.2. TDP features

TDPs are a set of broadband features with physical meanings, whose definition is as (Vidaurre et al., 2009).

(2) $$\begin{array}{l} TDP^{\left(p\right)}=\log(\text{var}(\frac{{\text{d}}^{p}\text{x}\left(t\right)}{\text{d}t^{p}})),\;p=0,1,2,\ldots , \end{array}$$

in which we apply logarithm to regulate the TDPs' distribution to Gaussian approximately (Vidaurre et al., 2009) to fit the LDA classifier (Müller et al., 2004).

Three TDPs are used in this paper to extract EEG features in both temporal and frequency domain, that is, **f**^*TDP*^ = [*TDP*^(0)^, *TDP*^(1)^, *TDP*^(2)^]. Typically, *TDP*^(0)^ depicts the EEG character in terms of amplitude, *TDP*^(1)^ can be interpreted as a kind of EEG pattern in terms of high frequency (mainly the beta band), and *TDP*^(2)^ carries the essential information of the change in frequency (Vidaurre et al., 2009).

#### 3.3.3. Feature compressing and data representation

With SP or TDP features extracted from all channels of all trials, we collect all features of training trials (with $\bar{N}$ trials) as a feature pool (${\left\{{\text{f}}_{i}\right\}}_{i=1}^{\bar{N}\times C\times 11}$ for SP features or ${\left\{{\text{f}}_{i}\right\}}_{i=1}^{\bar{N}\times C\times 3}$ for TDP features), abandoning its trial and channel information. Then, the classical *k*-means is used as an example to cluster the above EEG features. Likewise, almost all of data clustering approaches, such as spectral clustering and AP clustering, are acceptable to replace it.

In this paper, we utilize *k*-means clustering to partition $\bar{N}\times C\times 11$ or $\bar{N}\times C\times 3$ features into $K\left(\leq \bar{N}\right)$ sets ℂ = {ℂ_1_, ℂ_2_, …, ℂ_*K*_} in order to minimize the within-cluster sum of squares (WCSS). Formally, the object of *k*-means is to find:

(3) $$\begin{array}{l} \underset{\mathbb{C}}{\mathrm{argmin}}\overset{K}{\underset{j=1}{\sum }}\underset{i\in {\mathbb{C}}_{j}}{\sum }||{\text{f}}_{i}-\mu_{j}|{|}^{2}, \end{array}$$

where μ_*j*_ is the mean of features in ℂ_*j*_. This is equivalent to minimizing the pairwise squared deviations of features in the same cluster:

(4) $$\begin{array}{l} \underset{\mathbb{C}}{\mathrm{argmin}}\overset{K}{\underset{j=1}{\sum }}\frac{1}{2|{\mathbb{C}}_{j}|}\underset{i,i^{\prime }\in {\mathbb{C}}_{j}}{\sum }||{\text{f}}_{i}-{\text{f}}_{i^{\prime }}|{|}^{2} \end{array}$$

After dividing training features into *K* clusters using standard Lloyd's algorithm (Lloyd, 1982), we can replace feature vector **f**_*i*_ with its corresponding cluster signature *j* if *i*∈ℂ_*j*_. In this way, training signals can be represented as a matrix ${\tilde{\text{X}}}^{\bar{N}\times C}$.

#### 3.3.4. Channel ranking

After representing training trials as a normal data matrix $\tilde{\text{X}}$ with its rows and columns indicating trials and channels, we take RFS, RUFS and SSLSR as examples to rank and select EEG channels. The main strength of these methods is that they could find the optimal channel subset with the strict convergence in theory. Similarly, researchers can use other appropriate feature selection algorithms to rank channels.

In this section, $\tilde{\text{Y}}\in {\left\{0,1\right\}}^{\bar{N}\times L}$ is the class label indicator matrix (${\tilde{\text{Y}}}_{il}=1$ if the *i*-th sample belongs to the *l*-th class, otherwise, ${\tilde{\text{Y}}}_{il}=0$), and **W** ∈ ℝ^*C*×*L*^ is the weight matrix of channels. After obtaining **W**, we rank all channels according to their weights, which are computed as ||**W**_(*i*,:)_||_2_.

##### 3.3.4.1. Ranking based on RFS

RFS is an efficient and robust feature selection by emphasizing *l*_2, 1_-norm minimization on both the loss function and the regularization term (Nie et al., 2010). The objective of RFS is

(5) $$\begin{array}{l} \underset{\text{W}}{\min}J\left(\text{W}\right)=||\tilde{\text{X}}\text{W}-\tilde{\text{Y}}|{|}_{2,1}+\alpha ||\text{W}|{|}_{2,1}. \end{array}$$

Note that Equation (5) is equivalent to

(6) $$\begin{array}{l} \underset{\text{W},\text{E}}{\min}||\text{E}|{|}_{2,1}+||\text{W}|{|}_{2,1},\text{ s}.\text{t}.\;\tilde{\text{X}}\text{W}+\alpha \text{E}=\tilde{\text{Y}}. \end{array}$$

By setting $\text{A}=\left[\tilde{\text{X}}\;\alpha \text{I}\right]\in {\mathbb{R}}^{\bar{N}\times \left(C+\bar{N}\right)}$ and $\text{U}=[\begin{array}{c} \text{W}\\ \text{E} \end{array}]\in {\mathbb{R}}^{\left(C+\bar{N}\right)\times L}$, we can reformulate the objective of RFS as

(7) $$\begin{array}{l} \underset{\text{U}}{\min}||\text{U}|{|}_{2,1}+||\text{W}|{|}_{2,1},\text{ s}.\text{t}.\;\text{A}\text{U}=\tilde{\text{Y}}. \end{array}$$

Then, the optimal **U** is gained by setting the derivative of the Lagrangian function of Equation (7) on **U** to zeros, that is

(8) $$\begin{array}{l} \text{U}={\text{D}}^{-1}{\text{A}}^{T}{\left(\text{A}{\text{D}}^{-1}{\text{A}}^{T}\right)}^{-1}\tilde{\text{Y}}, \end{array}$$

where **D** is a diagonal matrix with the *i*-th diagonal element as ${\text{D}}_{ii}=\frac{1}{2||{\text{U}}_{\left(i,:\right)}|{|}_{2}}$.

The pseudo codes of RFS is given in Algorithm 1.

**Table:** Algorithm 1 Pseudo codes of RFS

**Input:**
EEG data matrices ${\tilde{\text{X}}}^{\bar{N}\times C}$, ${\tilde{\text{Y}}}^{\bar{N}\times L}$ and hyper-parameter α.
**Output:**
Channel weight matrix **W**.
1: Set *iter* = 0, $\text{A}=\left[\tilde{\text{X}}\;\alpha \text{I}\right]$ and initialize ${\text{D}}_{0}^{\left(C+\bar{N}\right)\times \left(C+\bar{N}\right)}$ as an identity matrix.
2: **repeat**
3: Calculate ${\text{U}}_{iter+1}={\text{D}}_{iter}^{-1}{\text{A}}^{T}{\left(\text{A}{\text{D}}_{iter}^{-1}{\text{A}}^{T}\right)}^{-1}\tilde{\text{Y}}$.
4: Update **D**_*iter*+1_, where the *i*-th diagonal element as ${\text{D}}_{ii}=\frac{1}{2||{\text{U}}_{\left(i,:\right)}|{|}_{2}}$.
5: iter = iter + 1.
6: **until** *Converges*

The theoretical proof of the convergence of Algorithm 1 can be found in Nie et al. (2010).

##### 3.3.4.2. Ranking based on RUFS

RUFS utilizes *l*_2, 1_-norm minimization on processes of both label learning and feature learning to effectively handle outliers and noise in the data, as well as reduce redundant or irrelevant channels. The objective of RUFS is often written as

(9) $$\begin{array}{l} \begin{array}{cc} \underset{\text{F},\text{G},\text{W}}{\min} & ||\tilde{\text{X}}-\text{G}\text{F}|{|}_{2,1}+\beta \text{trace}\left({\text{G}}^{T}\text{L}\text{G}\right)\\ +\gamma ||\tilde{\text{X}}\text{W}-\text{G}|{|}_{2,1}+\eta ||\text{W}|{|}_{2,1},\\ \text{s.t. } & \text{G}\in {\mathbb{R}}_{+}^{\bar{N}\times L},{\text{G}}^{T}\text{G}=\text{I},\\ \text{F}\in {\mathbb{R}}_{+}^{L\times C},\text{W}\in {\mathbb{R}}_{+}^{C\times L}, \end{array} \end{array}$$

where **L** is the normalized graph Laplacian matrix commonly used in unsupervised learning (Li et al., 2012).

Then, the optimal **W** is obtained by using a limited-memory BFGS (Nocedal, 1980) and BMLVM (Benson and Moré, 2001) based alternating iterative algorithm, which can be summarized in Algorithm 2.

**Table:** Algorithm 2 Pseudo codes of RUFS

**Input:**
EEG data matrix ${\tilde{\text{X}}}^{\bar{N}\times C}$ and hyper-parameters β,γ,η.
**Output:**
Channel weight matrix **W**.
1: Set *iter* = 0 and initialize **G**_0_, ${\text{F}}_{0}\leftarrow {\left[{\left({\text{G}}^{T}\text{G}\right)}^{-1}{\text{G}}^{T}\tilde{\text{X}}\right]}_{+}$, **W**_0_.
2: **repeat**
3: Fixing **G**_*iter*_, compute **W**_*iter*+1_ from L-BFGS algorithm given **G**_*iter*_, **W**_*iter*_, β and γ.
4: Fixing **F**_*iter*_ and **W**_*iter*+1_, compute **G**_*iter*+1_ from BMLVM algorithm given **G**_*iter*_, **F**_*iter*_, β and η.
5: Fixing **G**_*iter*+1_, compute **F**_*iter*+1_ from BMLVM algorithm given **G**_*iter*+1_, **F**_*iter*_.
6: iter = iter + 1.
7: **until** *Converges*

The theoretical proof of the convergence of Algorithm 2 is referenced in Qian and Zhai (2013) and Bezdek and Hathaway (2003).

##### 3.3.4.3. Ranking based on SSLSR

SSLSR is an effective greedy algorithm to directly handle the challenging *l*_2, 0_-norm based structural sparse least square regression, which has the objective

(10) $$\begin{array}{l} \underset{\text{W}}{\min}L\left(\text{W}\right):\underset{\text{W}}{\min}\frac{1}{\bar{N}}||\tilde{\text{X}}\text{W}-\tilde{\text{Y}}|{|}_{2}^{2},\text{s.t.}||\text{W}|{|}_{r,0}\leq \bar{C}, \end{array}$$

where $\bar{C}$ is the number of selected channels.

Start with **W**_0_ = **0**, SSLSR develops an alternating forward and backward strategy to pick and eliminate (if possible) a channel in each iteration.

Suppose at the beginning of the *iter*-th iteration, the selected channel set is 𝔽^*iter*^. Then $\forall i\in {𝔽}^{iter},{\text{W}}_{\left(i,:\right)}^{iter}\neq 0$. In the forward step, we need to find the channel which reduces the loss most, i.e., find *i* ∉ 𝔽^*iter*^ and θ∈ℝ^*L*×1^ such that

(11) $$\begin{array}{l} \underset{i\notin {𝔽}^{iter}}{\min}\underset{\theta }{\min}\;L\left({\text{W}}^{iter}+{\text{e}}_{i}\theta^{T}\right), \end{array}$$

where ${\text{e}}_{i}\in {\mathbb{R}}^{C\times 1}$ is the vector of zeros, except for the *i*-component which is one. θ is the optimal weight vector for one channel.

Since $L\left({\text{W}}^{iter}+{\text{e}}_{i}\theta^{T}\right)$ is convex in terms of θ^*T*^, the optimal θ for *i* ∉ 𝔽^*iter*^ can be gained by setting $\partial L\left({\text{W}}^{iter}+{\text{e}}_{i}\theta^{T}\right)/\partial \theta =0$, that is,

(12) $$\begin{array}{l} \theta ={\left(\tilde{\text{Y}}-\tilde{\text{X}}{\text{W}}^{iter}\right)}^{T}{\tilde{\text{X}}}_{\left(:,i\right)}/||{\tilde{\text{X}}}_{\left(:,i\right)}|{|}_{2}^{2}. \end{array}$$

Thus, we have

(13) $$\begin{array}{l} \left[i^{iter},\theta^{iter}\right]=\underset{i\notin {𝔽}^{iter}}{\mathrm{argmin}}\underset{\theta }{\mathrm{argmin}}\;L\left({\text{W}}^{iter}+{\text{e}}_{i}\theta^{T}\right). \end{array}$$

Before beginning the backward step, 𝔽 and **W** are updated as 𝔽^*iter*+1^ = 𝔽^*iter*^∪{*i*^*iter*^} and ${\text{W}}^{iter+1}={\text{W}}^{iter}+{\text{e}}_{i^{iter}}\theta^{iterT}$ respectively.

In the backward step, the channel with the least contribution to reducing the loss is selected and we need to determine whether to remove it according to a specific criterion. That is to say, we need firstly to find

(14) $$\begin{array}{l} j^{iter}=\underset{j\in {𝔽}^{iter}}{\mathrm{argmin}}\;L\left({\text{W}}^{iter}-{\text{e}}_{j}{\text{W}}_{\left(j,:\right)}^{iter}\right)-L\left({\text{W}}^{iter}\right), \end{array}$$

which is equivalent to finding *j* ∈ 𝔽^*iter*^ such that

(15) $$\begin{array}{l} \underset{j\in {𝔽}^{iter}}{\min}\;L\left({\text{W}}^{iter}-{\text{e}}_{j}{\text{W}}_{\left(j,:\right)}^{iter}\right). \end{array}$$

The eliminating criterion relies on the decrease and increase of the loss in forward and backward steps. Specifically, if and only if

(16) $$\begin{array}{l} \Delta_{+}^{iter}<\nu \Delta_{-}^{iter}, \end{array}$$

the *j*^*iter*^-th channel can be removed from 𝔽, where ν∈(0, 1) is a hyper-parameter, $\Delta_{-}^{iter}=L\left({\text{W}}^{iter}\right)-L\left({\text{W}}^{iter+1}\right)$ and $\Delta_{+}^{iter}=L\left({\text{W}}^{iter}-{\text{e}}_{j^{iter}}{\text{W}}_{\left(j^{iter},:\right)}^{iter}\right)-L\left({\text{W}}^{iter}\right)$.

The pseudo codes of SSLSR are as given in Algorithm 3 and Algorithm 4.

**Table:** Algorithm 3 Pseudo codes of SSLSR

**Input:**
EEG data matrix ${\tilde{\text{X}}}^{\bar{N}\times C}$ and class label indicator matrix ${\tilde{\text{Y}}}^{\bar{N}\times L}$, number of selected channels $\bar{C}$ and number of maximum iteration *MI*.
**Output:**
Selected feature index set 𝔽.
1: Initialize 𝔽^0^ = [ ] **W**^0^ = **0**^*C*×*L*^, *iter* = 0.
2: **repeat**
3: *% forward*
4: Find $\left[i^{iter},\theta^{iter}\right]=\underset{i\notin {𝔽}^{iter}}{\mathrm{argmin}}\underset{\theta }{\mathrm{argmin}}\;L\left({\text{W}}^{iter}+{\text{e}}_{i}\theta^{T}\right)$ by **Algorithm** **4**.
5: Update 𝔽^*iter*+1^ = 𝔽^*iter*^∪{*i*^*iter*^}.
6: Let ${\text{W}}^{iter+1}={\text{W}}^{iter}+{\text{e}}_{i^{iter}}\theta^{iterT}$.
7: Compute $\Delta_{-}=L\left({\text{W}}^{iter}\right)-L\left({\text{W}}^{iter+1}\right)$.
8: **if** Δ_−_ ≤ ϵ **then**
9: **break**
10: **end if**
11: Set *iter* = *iter*+1.
12: *% backward*
13: Find $j^{iter}=\underset{j\in {𝔽}^{iter}}{\mathrm{argmin}}\;L\left({\text{W}}^{iter}-{\text{e}}_{j}{\text{W}}_{\left(j,:\right)}^{iter}\right)$.
14: Compute $\Delta_{+}=L\left({\text{W}}^{iter}-{\text{e}}_{j^{iter}}{\text{W}}_{\left(j^{iter},:\right)}^{iter}\right)-L\left({\text{W}}^{iter}\right)$.
15: **if** Δ_+_≥νΔ_−_ **then**
16: **continue**.
17: **end if**
18: Set *iter* = *iter*−1
19: Update 𝔽^*iter*^ = 𝔽^*iter*+1^−{*j*^*iter*+1^}
20: Let ${\text{W}}^{iter}={\text{W}}^{iter+1}-{\text{e}}_{j^{iter+1}}{\text{W}}_{\left(j^{iter+1},:\right)}^{iter}$
21: **until** $|𝔽|=\bar{N}$ or *iter* = *MI*

**Table:** Algorithm 4 Pseudo codes of the forward step of SSLSR

**Input:**
EEG data matrices ${\tilde{\text{X}}}^{\bar{N}\times C}$ and class label indicator matrix ${\tilde{\text{Y}}}^{\bar{N}\times L}$, selected channel set 𝔽^*iter*^ and channel weight matrix **W**^*iter*^.
**Output:**
*i*^*iter*^ and θ^*iter*^.
1: Initialize $L_{min}=+\infty$ and $\text{T}=\text{Y}-{\text{F}}^{iter}=\text{Y}-\underset{i\in {𝔽}^{iter}}{\sum }{\tilde{\text{X}}}_{\left(:,i\right)}{\text{W}}_{\left(i,:\right)}^{iter}$
2: **for all** *i* ∉ 𝔽^*iter*^ **do**
3: Calculate $\theta =\frac{1}{||{\tilde{\text{X}}}_{\left(:,i\right)}|{|}_{2}^{2}}{\text{T}}^{T}{\tilde{\text{X}}}_{\left(:,i\right)}$
4: **if** ($L\left({\text{W}}^{iter}+{\text{e}}_{i}\theta^{T}\right)<L_{min}$) **then**
5: Update $L_{min}=L\left({\text{W}}^{iter}+{\text{e}}_{i}\theta^{T}\right)$
6: Set *i*^*iter*^ = *i* and θ^*iter*^ = θ
7: **end if**
8: **end for**

The theoretical proof of the convergence of Algorithm 3 can be found in Han et al. (2015a).

#### 3.3.5. Pseudo codes of the framework

The flowchart and pseudo codes of the proposed framework are summarized in Figure 1 and Algorithm 5.

**Table:** Algorithm 5 Pseudo codes of the proposed FCCR

**Input:**
*N* EEG data matrices $X_{1}^{M\times C}$, $X_{2}^{M\times C}$,…, and $X_{N}^{M\times C}$, hyper-parameter α, β, γ, η, ν.
**Output:**
*N*-dimensional predicted label vector $\tilde{\text{y}}$.
1: EEG data pre-processing.
2: EEG feature extraction channel-wise according to Section 3.3.2.
3: Partition all trials into training and testing sets.
4: Cluster features of training trials using *k*-means.
5: EEG data representation.
6: Channel ranking and selection by **Algorithm** **1**, **Algorithm** **2**, or **Algorithm** **3**.
7: Train LDA classifier using training trials with selected channels.
8: Predict the label vector of testing trails using trained LDA.

## 4. Results

We refer to our methods based on RFS, RUFS, and SSLSR as FCCR1, FCCR2, and FCCR3, respectively, and compare them with some baselines. In this section, we present the experimental setup, followed by the classification results.

### 4.1. Experimental setup

In this section, the baselines, metrics and other experimental settings are given in sequence.

#### 4.1.1. Baselines

To validate the effectiveness of FCCRs, we compare them with the following baselines, including three kinds of static channel subset and three kinds of channel selection with training algorithms.

1. **All channels**: Signals of all available channels are used for EEG classification;
2. **3C channel s**: Signals of C3, Cz, and C4 are used for EEG classification;
3. **MEMD+STFT** (Bashar et al., 2016): Features extracted based on multivariate empirical mode decomposition and short time Fourier transform are used for EEG classification;
4. **gsBLDA** (Yu et al., 2015): Signals of channels selected based on group sparse Bayesian linear discriminant analysis are used for EEG classification;
5. **MRCS** (Zhang et al., 2016): Signals of channels selected by combining ReliefF and SVM are used for EEG classification;
6. **NSGA-II** (Kee et al., 2015): Signals of channels selected by a multi-objective genetic algorithm, i.e., NSGA-II, are used for EEG classification.

#### 4.1.2. Metrics

Following previous researches, we employ the classification accuracy and F1 score to evaluate the performance of these methods.

Accuracy discovers the one-to-one relationship between predicted and ground truth labels. Denoting ${\tilde{\text{y}}}_{i}$ and **y**_*i*_ as the predicted result and the ground truth label of a trial **X**_*i*_ respectively, we can compute accuracy as follows.

(17) $$\begin{array}{l} Accuracy=\frac{1}{n}\overset{n}{\underset{i=1}{\sum }}\delta \left({\text{y}}_{i},\;{\tilde{\text{y}}}_{i}\right)\times 100\%, \end{array}$$

where *n* is the total number of trials and δ(*x, y*) is the delta function that equals 1 if *x* = *y* and 0 otherwise. A larger accuracy indicates a better performance.

F1 score is another widely used measure in evaluating the performance of classification. It is the harmonic mean of precision and recall,

(18) $$\begin{array}{l} F1=2\cdot \frac{Precision\times Recall}{Precision+Recall}\times 100\%. \end{array}$$

Again, a larger F1 indicates a better performance.

#### 4.1.3. Other settings

It is universally acknowledged that the performance of most algorithms is dependent on the hyper-parameters they contain. Therefore, we set some parameters in advance.

In the feature extraction process, for all methods except for MEMD+STFT, the SP features are extracted using the BCI2000 software tools (http://www.bci2000.org), with parameters as shown in Table 2 according to Krusienski et al. (2006), McFarland and Wolpaw (2008). Three TDPS, i.e., *TDP*^(0)^, *TDP*^(1)^, and *TDP*^(2)^ are extracted in this study. For MEMD+STFT, the hyper-parameters we used are identical to the original work (Bashar et al., 2016).

**Table 2:** Parameters used to extract SP features by BCI2000.

**Model order**	**First bin center**	**Last bin center**	**Bin width**	**Evaluations per bin**
16	0 Hz	30 Hz	3 Hz	15

In the feature clustering process, for our FCCRs, the number of clusters is determined using the "grid-search" strategy, i.e., from 2 to 8 with a step of 2, 10 to 90 with a step of 10, and 100 to 700 with a step of 200 on each subject, using the litekmeans algorithm.

In the channel ranking process, for gsBLDA, the stop criterion is whether the max iteration beyond 10 or the change of α or β is smaller than 0.01. For MRCS, the number of nearest neighbors *k* = 10, the maximum iteration number 50, ϵ = 0.01, and 10-fold cross-validation is used to evaluate the average classification accuracy when the top-n channels are used. For NSGA-II, the population is 300, the maximum generation is 200, and the 10-fold cross-validation accuracy is also utilized to estimate the first objective. The only difference from Kee et al. (2015) and Zhang et al. (2016) is that LDA, instead of the library for support vector machines (LIBSVM), is employed inside the channel selection procedure to maintain consistence with the following classification process.

In the trial classification process, 5 × 5 cross-validation on the LDA classifier is used throughout this study [except for MEMD+STFT, in which we use k nearest neighbor (kNN) according to Bashar et al., 2016]; i.e., five times for each dataset, we partition all the trials into five sets and choose four of them as the training sets, with the last set as the testing set. It should be noted that the training and testing sets are identical for all methods. For gsBLDA, MRCS, FCCR1, FCCR2, and FCCR3, the numbers of selected channels range from 1 to 118 (DS1) or 59 (DS2) with an incremental step of 1, and the numbers of the channels selected in NSGA-II are set according to the population with the best fitness.

### 4.2. Experimental results

To examine the effectiveness of the proposed framework, the classification experimental results are given and analyzed in this section. Because of the limited length of this paper, for some experiments related to subjects and features, we randomly partitioned the results for all subjects into different experiments, and show them in Figures 3–5. Thus, all nine subjects and two features are covered, if we consider these experimental results together.

#### 4.2.1. Classification performance comparison

We summarize the classification results of different methods using the two real world MI-EEG datasets in Tables 3–6.

**Table 3:** The mean classification accuracy (%) of nine methods on DS1.

**With the SP feature**	**aa**	**al**	**av**	**aw**	**ay**	**mean**
All channels	71.43 ± 4.37	83.57 ± 5.56	65.71 ± 8.41	76.07 ± 4.11	78.57 ± 3.34	75.07
3C channels	69.29 ± 5.84	**91.07** ± **2.19**	58.93 ± 3.79	79.29 ± 2.99	85.36 ± 6.61	76.79
MEMD + STFT	50.36 ± 5.11	54.64 ± 2.04	50.36 ± 5.42	39.29 ± 6.06	53.93 ± 8.32	49.71
gsBLDA	71.79(83)±4.26	87.86(42)±3.43	65.71(115)±7.08	77.86(91)±5.73	80.36(112)±4.55	76.71
MRCS	75.00(94)±1.26	84.64(89)±5.45	66.07(115)±7.14	77.50(94)±2.71	79.64(86)±7.21	76.57
NSGA-II	75.00(58)±5.50	86.43(55)±5.30	68.57(55)±6.87	80.36(58)±3.79	81.79(36)±5.56	78.43
FCCR1	75.36(82)±3.79	88.21(43)±5.14	**70.36 (11)** ± **9.57**	79.64(82)±1.60	86.43(4)±4.48	80.00
FCCR2	**80.00 (5)** ± **6.39**	90.71(80)±1.26	69.64(85)±4.07	**80.36 (97)** ± **4.82**	**88.93 (4)** ± **5.14**	**81.93**
FCCR3	76.79(7)±3.79	86.79(66)±2.04	68.57(72)±7.10	80.00(79)±3.87	86.79(4)±4.11	79.79
**With the TDP feature**	**aa**	**al**	**av**	**aw**	**ay**	**mean**
All channels	60.71 ± 3.57	85.00 ± 3.70	62.86 ± 7.19	68.21 ± 6.61	73.57 ± 3.87	70.07
3C channels	66.07 ± 10.02	91.43 ± 2.65	62.86 ± 3.87	78.21 ± 4.26	88.21 ± 5.73	77.36
MEMD + STFT	50.36 ± 5.11	54.64 ± 2.04	50.36 ± 5.42	39.29 ± 6.06	53.93 ± 8.32	49.71
gsBLDA	71.07(19)±5.56	94.29(17)±2.93	68.57(22)±5.14	80.36(14)±5.21	87.86(21)±6.11	80.43
MRCS	64.64(113)±2.93	87.86(38)±7.30	65.00(6)±5.14	76.43(20)±5.56	81.07(33)±6.00	75.00
NSGA-II	69.29(34)±6.61	94.64(26)±2.53	64.29(37)±4.19	84.64(33)±5.14	88.57(27)±4.82	80.29
FCCR1	75.00(15)±5.59	93.93(19)±3.91	**69.64 (14)** ± **5.30**	84.64(33)±6.36	88.57(26)±3.19	82.36
FCCR2	**75.71 (11)** ± **7.32**	**95.36 (14)** ± **2.71**	68.21(48)±4.79	80.71(32)±4.89	90.00(20)±3.43	82.00
FCCR3	73.57(13)±7.72	94.64(14)±1.79	67.86(9)±7.14	**85.00 (29)** ± **5.59**	**91.79 (10)** ± **4.11**	**82.57**

*The number of selected channels (listed in the bracket) and the standard deviation are also given. The best performance is highlighted in bold*.

**Table 4:** The mean classification accuracy (%) of nine methods on DS2.

**With the SP feature**	**a**	**b**	**f**	**g**	**mean**
All channels	74.50 ± 3.26	67.00 ± 4.81	62.00 ± 4.81	72.00 ± 5.97	68.88
3C channels	69.00 ± 6.02	61.00 ± 3.79	65.00 ± 5.59	**77.50** ± **3.95**	68.13
MEMD + STFT	54.00 ± 4.18	51.00 ± 7.42	52.00 ± 5.70	49.00 ± 8.40	51.50
gsBLDA	74.50(59)±3.26	67.00(57)±6.22	70.50(2)±6.94	75.50(24)±3.26	71.88
MRCS	74.50(59)±3.26	67.00(59)±4.81	63.00(58)±4.11	76.00(36)±2.85	70.13
NSGA-II	74.50(25)±7.79	62.00(23)±2.74	68.00(9)±3.26	73.00(23)±3.26	69.38
FCCR1	76.50(7)±5.86	69.50(47)±3.06	72.00(5)±7.79	**77.50 (5)** ± **5.76**	73.87
FCCR2	**77.00 (5)** ± **5.76**	**71.00 (21)** ± **8.18**	**76.50 (6)** ± **9.62**	77.00(25)±6.85	**75.38**
FCCR3	76.50(42)±8.40	69.00(51)±7.20	70.00(7)±3.95	77.00(33)±6.47	73.13
**With the TDP feature**	**a**	**b**	**f**	**g**	**mean**
All channels	62.50 ± 10.90	60.00 ± 7.91	55.00 ± 9.84	60.50 ± 11.51	59.50
3C channels	71.00 ± 8.02	63.50 ± 5.76	68.00 ± 6.71	80.00 ± 1.77	70.63
MEMD + STFT	54.00 ± 4.18	51.00 ± 7.42	52.00 ± 5.70	49.00 ± 8.40	51.50
gsBLDA	80.50(16)±2.74	65.00(14)±14.47	74.00(11)±8.22	77.50(17)±6.37	74.25
MRCS	76.50(25)±8.22	66.50(15)±9.29	72.50(8)±7.07	73.00(26)±6.22	72.13
NSGA-II	78.50(12)±5.76	69.00(24)±7.62	78.00(11)±5.70	79.50(10)±8.37	76.25
FCCR1	**83.50 (14)** ± **2.85**	69.50(8)±12.94	78.50(22)±3.79	**83.50 (18)** ± **4.87**	78.75
FCCR2	81.00(29)±4.68	**72.50 (9)** ± **3.26**	**81.00 (9)** ± **4.81**	82.50(8)±7.20	**79.25**
FCCR3	81.00(16)±5.18	71.00(18)±6.98	80.50(21)±4.47	82.00(18)±5.42	78.63

*The number of selected channels (listed in the bracket) and the standard deviation are also given. The best performance is highlighted in bold*.

**Table 5:** The mean F1-score (%) of nine methods on DS1.

**With the SP feature**	**aa**	**al**	**av**	**aw**	**ay**	**mean**
All channels	72.41 ± 4.15	84.25 ± 5.36	65.64 ± 8.71	75.74 ± 4.36	78.91 ± 3.90	75.39
3C channels	68.32 ± 5.69	90.71 ± 2.25	58.42 ± 3.17	77.36 ± 3.91	84.30 ± 7.87	75.82
MEMD + STFT	44.89 ± 8.77	52.84 ± 5.08	52.31 ± 3.87	36.65 ± 8.26	48.04 ± 8.95	46.94
gsBLDA	73.18(95)±3.14	88.27(39)±2.53	65.91(115)±7.16	78.45(91)±5.25	80.79(112)±5.20	77.32
MRCS	75.43(94)±2.31	85.49(87)±6.29	65.83(117)±8.22	77.30(94)±3.61	80.53(86)±6.62	76.92
NSGA-II	75.17(47)±7.88	86.97(55)±4.97	**70.06 (55)** ± **6.54**	**80.73 (58)** ± **4.12**	81.50(36)±5.79	78.89
FCCR1	76.16(113)±4.69	88.61(43)±4.56	69.93(11)±8.90	79.86(82)±2.97	87.16(4)±7.20	80.34
FCCR2	**80.99 (5)** ± **5.68**	**91.18 (80)** ± **1.03**	69.66(60)±5.35	80.51(97)±4.85	**89.53 (4)** ± **3.59**	**82.37**
FCCR3	78.44(7)±3.06	87.55(66)±1.82	68.61(55)±4.04	79.65(79)±4.34	87.11(4)±4.00	80.27
**With the TDP feature**	**aa**	**al**	**av**	**aw**	**ay**	**mean**
All channels	58.52 ± 3.89	85.09 ± 3.75	62.61 ± 6.21	68.59 ± 7.62	73.29 ± 5.53	69.62
3C channels	66.94 ± 8.42	91.70 ± 2.42	63.49 ± 4.82	77.82 ± 6.03	88.76 ± 4.84	77.74
MEMD + STFT	44.89 ± 8.77	52.84 ± 5.08	52.31 ± 3.87	36.65 ± 8.26	48.04 ± 8.95	46.94
gsBLDA	71.89(13)±4.91	94.49(17)±2.66	68.51(22)±5.54	79.75(54)±6.53	87.92(21)±6.28	80.51
MRCS	64.09(113)±4.34	88.46(38)±6.40	67.62(6)±6.83	76.05(20)±5.81	81.49(33)±5.64	75.54
NSGA-II	69.36(34)±6.10	94.68(26)±2.48	65.21(28)±10.91	**84.91 (33)** ± **4.65**	88.73(27)±4.59	80.58
FCCR1	75.86(15)±4.86	94.09(33)±3.74	**69.86 (14)** ± **3.96**	84.67(33)±4.88	88.68(26)±1.48	**82.63**
FCCR2	**76.42 (11)** ± **6.31**	**95.55 (18)** ± **4.98**	69.60(48)±7.48	80.71(35)±4.62	90.00(20)±5.27	82.45
FCCR3	73.30(13)±8.48	94.85(32)±3.42	68.45(7)±3.79	84.57(29)±5.79	**91.77 (10)** ± **3.99**	82.59

*The number of selected channels (listed in the bracket) and the standard deviation are also given. The best performance is highlighted in bold*.

**Table 6:** The mean F1-score (%) of nine methods on DS2.

**With the SP feature**	**a**	**b**	**f**	**g**	**mean**
All channels	74.09 ± 2.60	67.95 ± 4.34	60.77 ± 8.73	71.51 ± 7.26	68.58
3C channels	66.70 ± 5.44	62.08 ± 3.37	62.65 ± 8.19	**76.57** ± **4.63**	67.00
MEMD + STFT	51.11 ± 2.99	40.23 ± 7.26	50.25 ± 9.65	47.93 ± 7.15	47.38
gsBLDA	74.09(59)±2.60	68.27(57)±5.93	69.10(2)±8.51	75.05(24)±5.18	71.63
MRCS	74.09(59)±2.60	68.24(58)±3.75	63.38(11)±5.77	75.56(36)±4.12	70.32
NSGA-II	74.15(25)±7.81	63.61(23)±3.66	66.14(9)±4.13	71.33(23)±2.53	68.81
FCCR1	75.49(7)±4.80	70.88(47)±5.29	72.59(5)±9.90	75.79(57)±7.91	73.69
FCCR2	75.85(5)±6.38	**72.44 (21)** ± **9.74**	**76.12 (6)** ± **10.31**	76.11(25)±5.34	**75.13**
FCCR3	**76.17 (46)** ± **6.13**	70.35(51)±5.71	70.59(7)±3.22	76.05(24)±6.48	73.29
**With the TDP feature**	**a**	**b**	**f**	**g**	**mean**
All channels	63.18 ± 9.86	58.52 ± 8.57	50.94 ± 16.99	61.42 ± 11.54	58.51
3C channels	68.87 ± 8.28	63.29 ± 5.21	67.31 ± 8.44	79.38 ± 1.24	69.71
MEMD + STFT	51.11 ± 2.99	40.23 ± 7.26	50.25 ± 9.65	47.93 ± 7.15	47.38
gsBLDA	78.99(16)±1.43	65.29(4)±5.16	74.77(11)±7.40	78.11(18)±3.32	74.29
MRCS	75.58(25)±10.68	65.94(15)±10.66	71.94(8)±8.50	70.81(23)±5.18	71.07
NSGA-II	78.29(12)±5.98	69.75(24)±7.83	78.47(11)±6.10	78.52(13)±7.37	76.26
FCCR1	**83.59 (14)** ± **3.41**	69.30(8)±14.41	78.17(22)±4.01	**82.88 (18)** ± **5.21**	78.48
FCCR2	80.09(34)±3.90	**72.81 (9)** ± **3.12**	**80.90 (9)** ± **4.37**	82.14(8)±5.37	**78.99**
FCCR3	79.87(16)±5.93	71.46(18)±6.76	80.35(21)±5.57	81.76(16)±4.49	78.36

*The number of selected channels (listed in the bracket) and the standard deviation are also given. The best performance is highlighted in bold*.

From these four tables, we make the following observations. First, the classification performance of the selected channel subsets is superior to All channels, demonstrating the practical significance of channel selection. For instance, as shown in Table 3, the best mean accuracies for the selected channels on DS1 are 81.93 and 82.57%; i.e., they are 9.14 and 17.8% better than the 75.07 and 70.07% for All channels, respectively.

Second, channel subsets selected through training often perform better than static channel subsets, as they verify the role of channel selection approaches using training. Taking Table 6 as an example, the best mean F1 scores on DS2 for training methods are 75.13 and 78.99%, which outperform the 67.00 and 69.71% of the static 3C channels by 12.1 and 13.3%, respectively.

Third, our proposed FCCRs usually outperform other channel selection methods on various subjects from different datasets. In terms of mean accuracies and F1 scores, our FCCRs consistently obtain the best performance. Specifically, of 36 results on nine subjects with two features in terms of two metrics, the FCCRs gain the 31 best results, occupying 86.1%.

Finally, our FCCRs can sometimes gain the best performance with fewer channels, indicating the effectiveness of our framework. Especially on subject ay with SP features, all three FCCRs obtain high accuracies and F1 scores with only four channels. In contrast, the fewest channels selected by other methods are 36 (NSGA-II), nine times the FCCRs' results.

**Figure 2:**
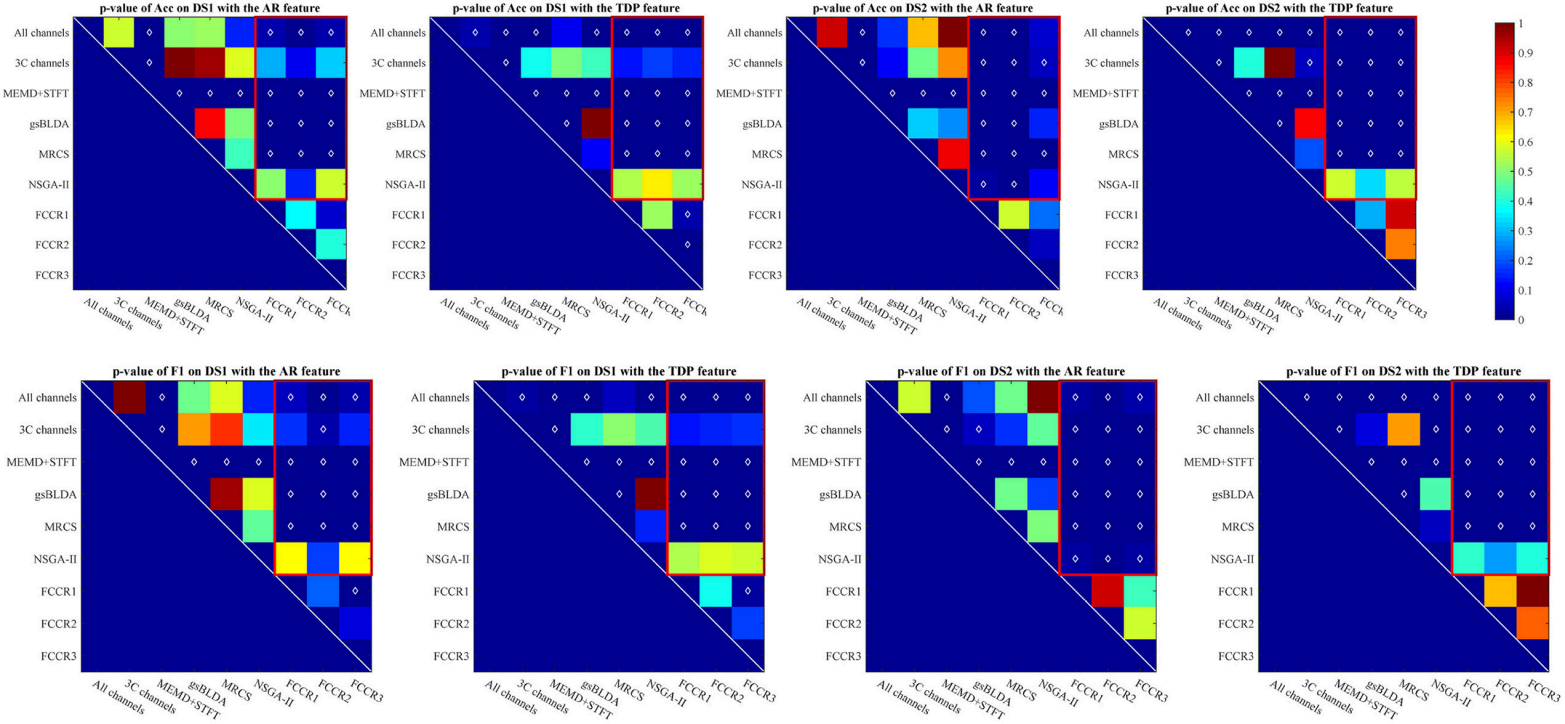
*p*-value for the improvements of nine methods on two datasets with SP or TDP features. The white diamonds at the center of each square denote a statistically significant improvement. Red rectangles mark the *p*-values of the proposed FCCRs relative to the baselines.

The *p*-values of one-way analysis of variance (ANOVA)^1^ statistical tests of the improvement for all methods are plotted in Figure 2, from which we can observe that the FCCRs obtain a significant improvement over the other methods in most situations. The three FCCRs have 18 improvements over six baselines for each panel; an average of 14 improvements is significant, occupying 77.8%.

**Figure 3:**
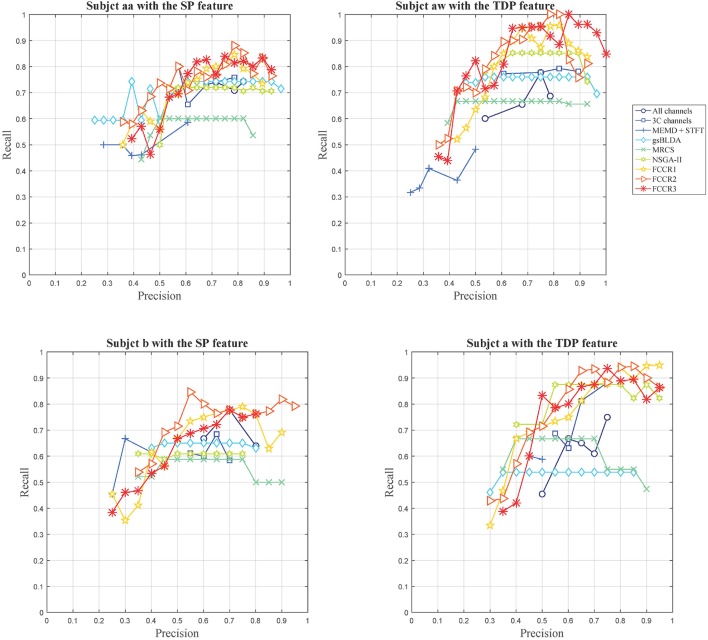
Precision-recall curves for nine methods on four subjects with SP or TDP features.

Except for the above, we plot the precision and recall (P-R) pairs for all methods in Figure 3, from which we can obtain other observations. First, some channel selection approaches may be limited in the recall, e.g., MRCS and NSGA-II on subject aa with SP features; gsBLDA, MRCS and NSGA-II on subject aw with TDP features; and gsBLDA on subject a with TDP features.

Second, our FCCRs can obtain a relatively high precision with acceptable recall. Specifically, on subject aw with TDP features, FCCR2, and FCCR3 both gain the recall of 1 and the precision larger than 0.8 at the same time, much better than the baselines.

Third, the P-R curve varies obviously with the subjects. For subjects aa and b with SP features, the recall of all the methods is below 0.9, and the precision is hardly beyond 0.9. Conversely, many markers are distributed in the area beyond 0.9 in terms of both precision and recall for subjects aw and a with TDP features.

#### 4.2.2. Selected channels comparison

**Figure 4:**
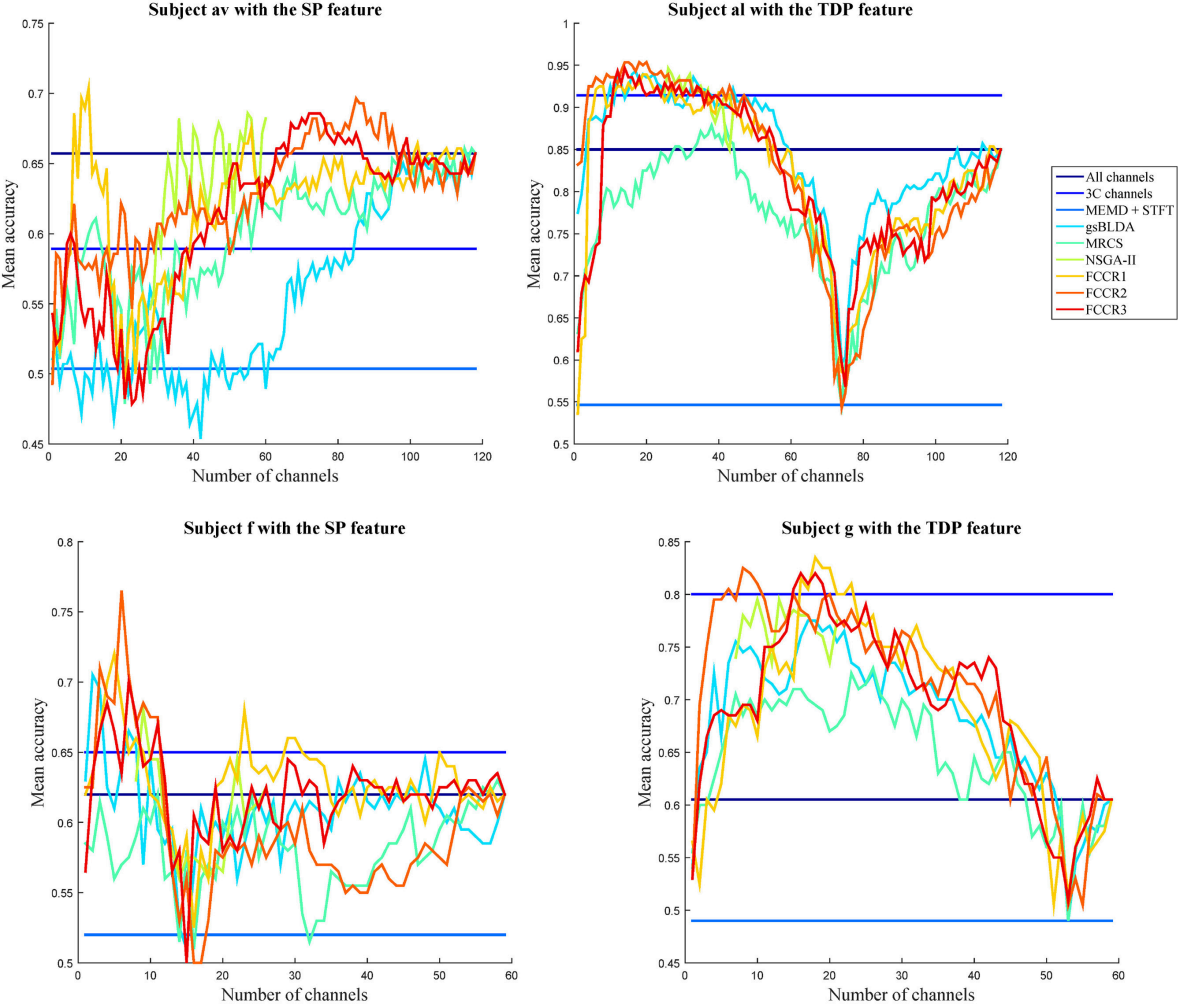
Average classification accuracy and F1 scores of nine algorithms with numbers of selected channels varying from one to all available in the step of one on subjects with different features.

To visually compare the performance of the different channel selection approaches, we plot the classification accuracies of nine methods with different numbers of selected channels on four subjects in Figure 4. The observations are as follows. First, almost all channel selection methods are effective, including 3C channels, gsBLDA, MRCS, NSGA-II and three FCCRs, since their accuracies with fewer selected channels can outperform the ones with all channels. Second, few channel selection results are superior to the ones with 3C channels. In fact, on subject g with TDP features, only the FCCRs outperform the 3C channels. Third, the proposed FCCRs can obtain the best performance compared with the other methods in most situations. For these four results, the best performance is always acquired by FCCRs. Fourth, our FCCRs are able to gain the highest accuracies with few channels, which is consistent with the above results.

**Figure 5:**
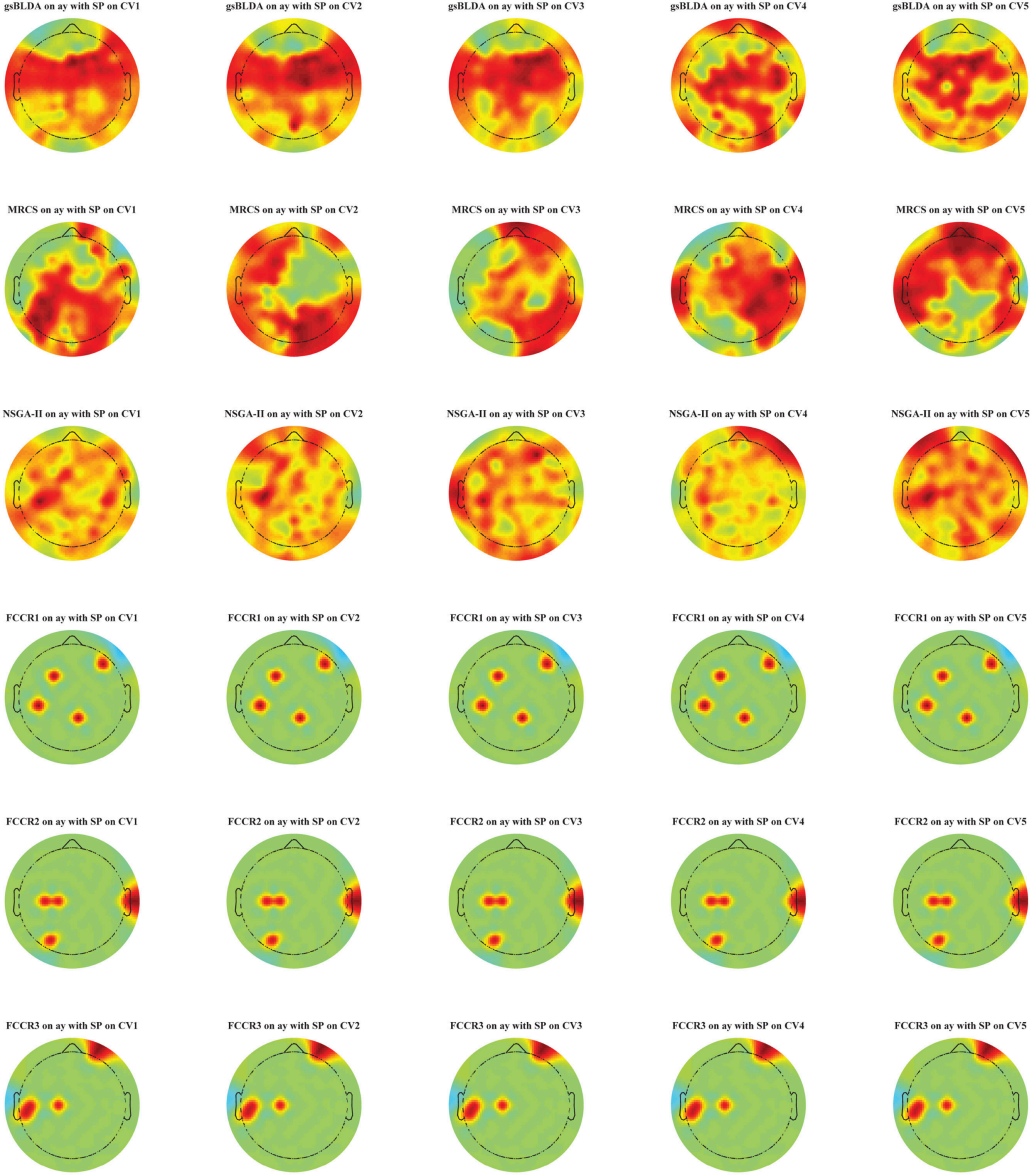
Topographical map of multichannel MI-EEG recordings for subject ay with the SP feature. Six rows indicate six methods, i.e., gsBLDA, MRCS, NSGA-II and our three proposed methods. Five columns indicate the five cross-validation. We only retain the channels included in the optimal channel subset. Red and blue represent the top and bottom rankings.

Additionally, in Figure 5, we display the selected channel subset on the subject's scalp with the top classification accuracies for subject ay, in which the channels ranked from top to bottom are indicated by the red to blue areas. It is clear that significant and informative channels relevant to the MI-EEG, i.e., channels in the motor-sensory cortex, are selected first by the proposed FCCRs. gsBLDA, MRCS and NSGA-II usually select some irrelevant or redundant channels, possibly having an adverse effect on their classification performance, which is consistent with the results in section 4.2.1 and Figure 4.

#### 4.2.3. Computational complexity comparison

In this subsection, we experimentally analyze the computational complexity of nine methods by comparing their average running time (ART) for nine subjects with two datasets, on a PC with a 4.0GHz Intel i-7 6700K CPU with 32 GB of memory.

**Figure 6:**
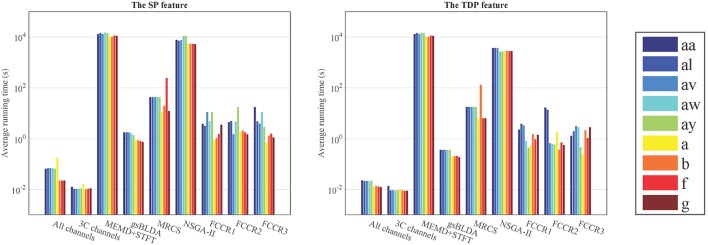
Average running time (s) of nine methods on nine subjects with the configurations with the best accuracies.

From Figure 6 and Table 7, we can observe that the ARTs of the FCCRs are longer than some approaches without training, including All channels and 3C channels. In addition, the fact that the FCCRs run faster than MRCS, NSGA-II, and MEMD+STFT demonstrates their efficiency. It is interesting that MEMD+STFT is unexpectedly slow, even though it has no channel selection. It is also clear that the ARTs of FCCRs are < 10 s on nine subjects, which is comparable with motor imagery paradigms, demonstrating their practical worth in real applications.

**Table 7:** Average running time (ART) measured in *s* of nine methods on two datasets.

**Method**	**DS1**		**DS2**		**Mean**
	**With the SP feature**	**With the TDP feature**	**With the SP feature**	**With the TDP feature**
All channels	1.086	0.253	0.685	0.153	0.544
3C channels	**1.031**	**0.242**	**0.637**	**0.149**	**0.515**
MEMD + STFT	13,949	13,949	10,752	10,752	12,351
gsBLDA	1.670	0.354	0.818	0.196	0.760
MRCS	43.31	17.78	71.42	37.76	42.57
NSGA-II	8,832	3,316	5,368	2,790	5,077
Proposed1	6.828	2.149	1.764	1.105	2.962
Proposed2	6.645	6.394	1.811	0.858	3.927
Proposed3	8.100	1.961	1.187	1.580	3.207

*The shortest ARTs are highlighted in bold*.

#### 4.2.4. Parameter sensitivity

**Figure 7:**
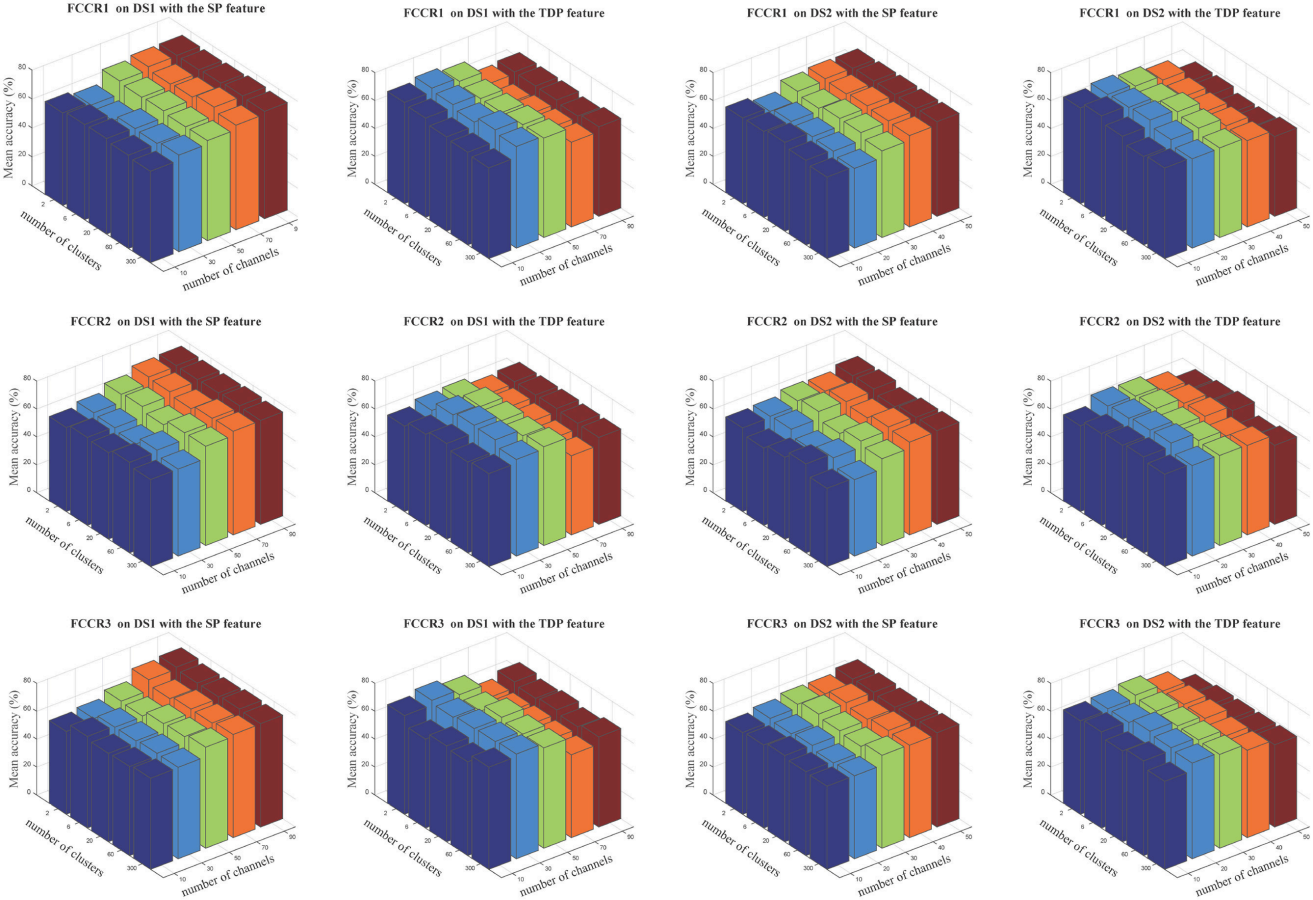
Average classification accuracies(%) of our three methods when the numbers of selected channels are 10, 30, 50, 70, and 90 for DS1 and 10, 20, 30, 40, and 50 for DS2, and the numbers of clusters are 2, 6, 20, 60, and 300. The three rows correspond to FCCR1, FCCR2 and FCCR3, respectively.

The insensitivity of our proposed framework to cluster numbers is demonstrated in Figure 7, in which we notice that cluster numbers exert a limited influence on classification accuracies in quite a large range, from 2 to 300.

#### 4.2.5. Comprehensive comparison

**Figure 8:**
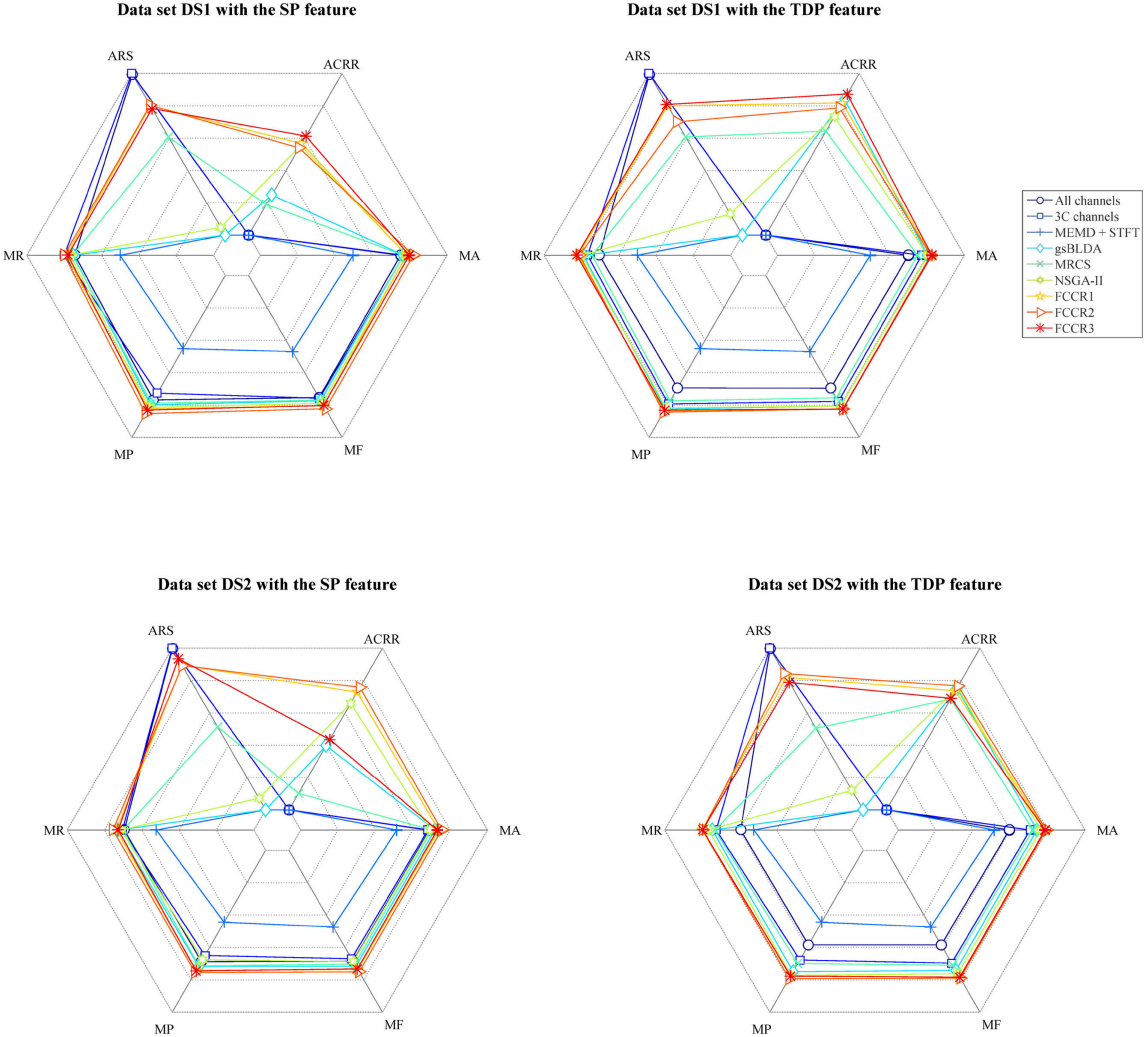
Mean accuracy, mean F1 score, mean precision, mean recall, average running speed and average channel reduction ratio for nine algorithms. Except for the average running speed, whose range varies according to the practical minimum and maximum values of the nine methods, the range of the other metrics is [0,1] ([0%,100%] for ACRR).

In addition to the above experiments, we comprehensively compare nine methods comprehensively in terms of six metrics, i.e., mean accuracy (MA), mean F1 score (MF), mean precision (MP), mean recall (MR), average running speed (ARS, number of trials divided by ART), and average channel reduction ratio (ACRR, ratio of number of removed channels to all channels), and show the results in Figure 8. In this figure, the inner hexagon indicates 0 for MA, MF, MP, and MR, the smallest ARS, and 0% for ACRR. The outer hexagon indicates 1 for MA, MF, MP, and MR, the largest ARS, and 100% for ACRR. Thus, for all results, more outside markers mean better performance.

It appears from Figure 8 that, for all data sets with SP or TDP features, the FCCRs are superior to other state-of-the-art methods in terms of almost all metrics, except for ARS.

## 5. Discussion

From the experimental results shown above, we find that our methods are conducive to EEG classification through feature compression, low-dimensional data representation, and convergent channel ranking. The primary reasons for the promising classification performance of our FCCRs include the following: (1) motor-sensory cortex signals are much more relevant to MI-EEG than signals of other cortexes; signals from redundant channels corresponding to unrelated cortexes may negatively influence the EEG classification performance; (2) informative channels often vary between subjects and the optimal channel subsets need to be found empirically; the intrinsic physiological and mental condition divergence among subjects during the experimental process is fairly large; (3) our FCCRs can probably select more pivotal channels and rank them in the top position, by virtue of their channel selection methods with high capability and strict convergence.

With regard to the selected channel comparison results, the primary explanations follow: (1) signals from irrelevant and redundant channels have a negative effect on classifying MI-EEG trials, as they introduce confusing information; (2) 3C channels, i.e., Cz, C3, and C4, are closely related to the motor-sensory cortex and pivotal for classifying motor imagery EEG signals; they are infrequently selected by some methods.

The computational complexity comparison results could be intuitively foreseen by comparing their flowcharts and procedures. The detailed explanation mainly includes the following: (1) the process of evaluating the channels' weights is unnecessary in methods without training, e.g., All channels and 3C channels; thus, their ARTs are small; (2) the FCCRs compress the feature vectors and exploit strictly convergent channel selection approaches to reduce the data scale and the period of evaluating the channels' weights; they thereby accelerate the EEG classification procedure and decrease the FCCRs' ARTs; (3) methods with embedded classifiers, e.g., MRCS and NSGA-II, need to estimate the channel subset and update the channel's weights in each iteration, leading to large ARTs. Additionally, MEMD+STFT has large ARTs because extracting the MEMD features is time consuming, especially when the total number of signal projections is beyond 200 in the MEMD algorithm.

For the parameter sensitivity results, the cluster numbers have limited influence on estimating the similarity among features and clustering-based representative signals. Therefore, changing the number of clusters does not significantly affect the following channel selection and classification.

From the comprehensive comparison results, we find that the FCCRs have some exciting advantages, including a small data scale, a strictly convergent procedure, short running time, and high classification accuracy. All of these visibly promote their comprehensive performance, which is helpful for implementing them with field programmable gate arrays (FPGAs) or digital signal processors (DSPs) in practical applications.

As shown in Figure 1, the main differences between the FCCRs and previous studies are feature compression and convergent channel ranking. On one hand, in current EEG feature extraction methods, spatial features, or features in the time and frequency domain, are always *directly* used to select channels, and even classify trials (Blankertz et al., 2007; Zhaoyang et al., 2016). From the perspective of classical pattern recognition, the essential role of the features depends on their similarities or differences, rather than the explicit numerical values themselves. Therefore, compressing features by clustering them and endowing them with signatures not only preserves the intrinsic similarities involved in the features, but also reduces the data scale. On the other hand, most existing channel selection methods carefully evaluate the channels' weights by *directly* classifying the signals that compose them (Kee et al., 2015; Zhang et al., 2016). Thus, their performance is classifier-dependent, lacking adaptability and support for unfamiliar situations. In most practical applications, classifiers are not fixed and should be flexibly chosen according to practical conditions. Moreover, ignoring the convergence of the selection methods may lead to unsatisfactory results, since the process of adding or removing channels is not controllable. Thus, it is better to leverage classifier-independent and performance-verified selection approaches with proven strict convergence to guarantee both the process and the result of the channel weights evaluation. Philosophically, some solutions have tortuous exteriors but are very rapid in many researching areas. By introducing feature compression and convergent channel selection, we propose the FCCRs as such a promising opportunity to classify multiple EEG signal types.

Inevitably, FCCRs still have some potential limitations to be overcome, which may bring a forth series of innovations and applications by integrating plenty of current and forthcoming signal processing and pattern recognition approaches in the future. Firstly, we will focus on the feature extraction and clustering methods for seizing the key information from the original EEG signals with low SNRs. Moreover, considering the ever-developing feature selection approaches, dynamic weighting techniques for evaluating channels are also among our directions for future research. In addition, deciding the optimal number of channels is still an open problem, for which we will attempt to propose a criterion in the near future.

## 6. Conclusion

In this paper, we present a novel EEG classification framework named FCCR, which introduces feature compression and normal signal representation between the traditional feature extraction and channel selection procedures. In addition, some strict convergent algorithms with theoretical proofs are leveraged to rank and select informative channels, thereby showing that our proposed framework is capable of classifying EEG signals with acceptable accuracy and speed. Extensive experimental results on two real world motor imagery EEG datasets validate the effectiveness and efficiency of the proposed framework, as compared with state-of-the-art methods.

## Author contributions

JH, JZ, and CW designed research. JH and YZ performed research. AK, GX, and HZ analyzed data and drafted parts of the first version of the manuscript. JH, HS, and JC wrote and revised the paper under the supervision of JZ and CW.

### Conflict of interest statement

The authors declare that the research was conducted in the absence of any commercial or financial relationships that could be construed as a potential conflict of interest.

## Footnotes

^1^To completely compare the methods with channel selection, we compute the *p*-values using the Acc or F1 across all 118(DS1) or 59(DS2) channels and five validations for gsBLDA, MRCS and the FCCRs. The results in Tables 3–6 are used to compute the *p*-values for other methods.

## Appendix

For any vector **x** ∈ ℝ^*n*×1^, the *l*_*p*_-norm is defined as

(A1) $$\begin{array}{l} ||\text{x}|{|}_{p}={\left(\overset{n}{\underset{i=1}{\sum }}|x_{i}{|}^{p}\right)}^{\frac{1}{p}}, \end{array}$$

where *x*_*i*_ is the *i*-th element of **x**. For any matrix **X** ∈ ℝ^*n*×*m*^, the *m*_*r*_ -norm and *m*_*r, s*_ -norm are defined as

(A2) $$\begin{array}{l} ||\text{X}|{|}_{r}={\left(\overset{n}{\underset{i=1}{\sum }}\overset{m}{\underset{j=1}{\sum }}|{\text{X}}_{ij}{|}^{r}\right)}^{\frac{1}{r}}, \end{array}$$

(A3) $$\begin{array}{l} ||\text{X}|{|}_{r,s}={\left(\overset{n}{\underset{i=1}{\sum }}{\left(\overset{m}{\underset{j=1}{\sum }}|{\text{X}}_{ij}{|}^{r}\right)}^{\frac{s}{r}}\right)}^{\frac{1}{s}}. \end{array}$$
